# Commentary: “Can Selective MHC Downregulation Explain the Specificity and Genetic Diversity of NK Cell Receptors?”

**DOI:** 10.3389/fimmu.2015.00444

**Published:** 2015-08-31

**Authors:** Philippe A. Robert

**Affiliations:** ^1^Department of Systems Immunology and Braunschweig Integrated Centre of Systems Biology, Helmholtz Centre for Infection Research, Braunschweig, Germany; ^2^Centre National de la Recherche Scientifique UMR5535, Institut de Génétique Moléculaire de Montpellier and Université de Montpellier, Montpellier, France

**Keywords:** iNKR, co-evolution, viral evasion strategies, computational model, population model

In Ref. (1) and in the study of interest (2), Carrillo-Bustamante et al. investigate *in silico* whether the diversity of inhibitory NK receptors (iNKRs) can be explained by viral evasion mechanisms involving MHC downregulation.

Inhibitory NK receptors on NK cells recognize Type I MHCs on healthy cells, maintaining NK cells in an inhibited state. Several viruses have the ability to downregulate MHC expression via different mechanisms (3), resulting in avoidance of CD8 T-cell-mediated toxicity but stimulating NK cells by decreasing their inhibition.

One would therefore expect that selective forces from the host side should select for a small set of iNKRs that can recognize all MHCs. iNKRs that are specific to particular MHC alleles would be poor detectors of viral invasion and would not be selected. It is thus striking to observe that human iNKRs alleles show the exact opposite trend, exhibiting both huge diversity and recognition of specific HLA alleles (4).

The authors consider two mechanisms that could account for this observation: (a) the viral expression of proteins mimicking MHCs epitopes (“decoys”) in addition to downregulation of all MHCs in Ref. (1) and (b) the ability to downregulate only a specific MHC locus, thus preserving the expression of the other locus in Ref. (2). To test their relative effect, the authors developed a population model of diploid individuals carrying both MHC and iNKR alleles, where viruses can spread randomly. Selective pressure is incorporated by assuming different survival rates of infected individuals depending on the recognition of this downregulation by iNKRs.

The configuration of MHCs and iNKRs within one individual is elegantly formulated and embodies, in the simplest way, the “core” hypotheses needed to simulate iNKR-mediated viral recognition (summarized in Figure 1).

**Figure 1:**
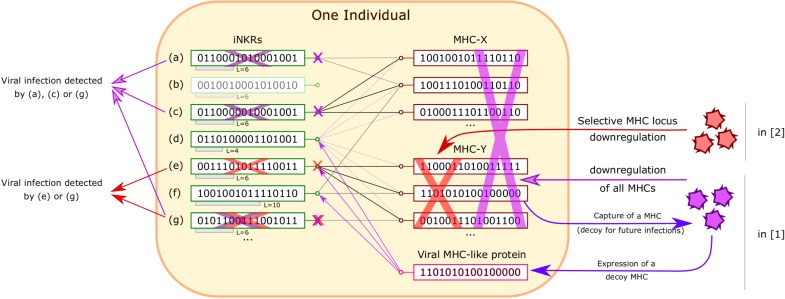
**Recognition of viral infection by iNKRs in Ref. (1, 2)**. Inside an individual, iNKRs (represented by binary code) recognize MHC alleles, provided that they share enough complementarity (relative to the sensitivity L of the iNKR). In this figure, recognition is represented as a match between iNKRs and MHCs. MHC downregulation is detected by an iNKR, provided that it loses all connections to MHCs. In Ref. (1), viruses are allowed to downregulate all MHCs and to express a viral protein that mimics a MHC (decoy). In this case, iNKRs recognizing only a few MHCs, such as (g) are advantageous because they are less easily lured by the decoy MHC. In Ref. (2), viruses can silence only MHCs from one locus (X or Y). iNKRs that are specific for all the MHCs from one locus but not from the other one, such as (c) and (e) are optimal (“excellent detectors”). In both cases, “degenerate” iNKRs recognizing all MHCs, such as (d) are ineffective and even deleterious in Ref. (1), as the virus can re-use them as decoys.

With the parameter values chosen by the authors, it is interesting to see that the iNKRs alleles emerging during evolution are not optimal but are instead diverse and redundant. For instance, in the case of specific MHC locus downregulation (2), only two iNKRs, each recognizing only MHCs from one locus but not the other one, would be sufficient to confer immunity against the virus. By contrast, the model shows that, even if some effective iNKRs are selected, they are outnumbered by suboptimal iNKRs. A weak point is the lack of sensitivity analysis for the population settings, and one could further use the model to assess which parameter values are critical and necessary for this diversity; for instance, by tuning the mutation rate versus selection pressure through including more or less virus infectivity in the model.

The authors characterized the selected iNKRs depending on how many MHCs from each locus that they recognize. Though the selected iNKRs are not all optimal, they cluster in groups, in which most iNKRs are more likely to recognize MHCs from one locus than the other, which is an intuitive prediction. It would be interesting to know how similar these iNKRs are, and whether they came from a common ancestor or emerged in parallel.

Although in Ref. (1), only one MHC locus was considered, in Ref. (2), the MHCs from the two loci were chosen to be dissimilar. They were generated at a limited hamming distance (“HD”) from two different seed sequences, making it easier for iNKRs to recognize MHCs from one locus but not the other when HD is low (i.e., iNKRs within a cluster are very similar to each other but different from those from the other cluster). Intuitively, the higher the HD, the harder it is for iNKRs to be efficient, because MHCs are harder to differentiate. It would suggest that MHCs need to be different enough between loci to allow for proper viral detection. Intriguingly, the sensitivity of selected iNKRs is independent of HD, suggesting the existence of an optimal sensitivity.

To sum up, this elegant formulation of iNKR evolution makes counter-intuitive predictions and raises new questions as well as possible further developments.

In order to account for the co-evolution of iNKRs, MHCs, and viruses, one might investigate an extended model in which not only iNKRs but also MHCs and the virus could mutate as well. It would be interesting to see if iNKRs can, in turn, exert a selective pressure on MHCs (e.g., by amplifying similarity within a MHC cluster). Additional selective pressures could emerge from the viruses. For instance, in the configuration with decoy viral molecules, it would be of interest to see how a virus evolves and mutates its decoy when facing selection pressure from a set of co-evolving iNKRs. This decoy would tend to be similar to all MHCs. In turn, to adapt, the iNKR pool would benefit by being as diverse as possible to avoid the existence of efficient decoys. This might also explain the extra diversity and the potential necessity for many iNKR loci. Finally, one could ask the model whether additional functions of iNKRs, such as recognition of microbial derivatives (5), could significantly impact their evolution.

## Conflict of Interest Statement

The author declares that the research was conducted in the absence of any commercial or financial relationships that could be construed as a potential conflict of interest.
